# Long‐Term Efficacy and Safety of Oleogel‐S10 (Birch Triterpenes) for Pediatric Patients With Epidermolysis Bullosa

**DOI:** 10.1111/pde.70224

**Published:** 2026-05-28

**Authors:** Eli Sprecher, Mauricio Torres‐Pradilla, Maria Florencia Fernandez, Christine Bodemer, Anna L. Bruckner, Silvia Maria de Macedo Barbosa, Andrea Diociaiuti, Anna E. Martinez, Francis Palisson, Miloš Nikolić, Asunción Vicente, Raúl de Lucas, Noemi Toiber Temin, Sandra Löwe, Johannes S. Kern, Dédée F. Murrell, the EASE Study Group

**Affiliations:** ^1^ Tel Aviv Sourasky Medical Center and Faculty of Medicine and Health Sciences, Tel Aviv University Tel Aviv Israel; ^2^ Department of Dermatology Hospital de San José, Fundación Universitaria de Ciencias de la Salud Bogotá Colombia; ^3^ Fundacion Respirar, Centro Medico Dra. De Salvo, Capital Federal Buenos Aires Argentina; ^4^ Department of Dermatology Expert Centre for Genodermatoses (MAGEC) Necker‐Enfants Malades Hospital, University Paris Centre, APHP Paris France; ^5^ Department of Dermatology University of Colorado School of Medicine Aurora Colorado USA; ^6^ Pain and Palliative Care Unit, Instituto da Criança, Hospital das Clínicas, Faculty of Medicine, Universidade de São Paulo São Paulo São Paulo Brazil; ^7^ Dermatology Unit and Genodermatosis Research Unit Translational Pediatrics and Clinical Genetics Research Area, Bambino Gesù Children's Hospital‐IRCCS Rome Italy; ^8^ Department of Dermatology Great Ormond Street Hospital London UK; ^9^ Department of Dermatology Faculty of Medicine, Clínica Alemana‐Universidad del Desarrollo, Santiago, Chile Fundación DEBRA Chile Santiago Chile; ^10^ Department of Dermatology and Venereology University of Belgrade School of Medicine; Clinic of Dermatology and Venereology, University Clinical Center of Serbia Belgrade Serbia; ^11^ Dermatology Unit, Hospital Sant Joan de Déu Barcelona Spain; ^12^ Dermatology Unit, Hospital Universitario La Paz Madrid Spain; ^13^ Chiesi GRD Clinical Development Toronto Canada; ^14^ Chiesi GRD Dublin Ireland; ^15^ The School of Translational Medicine, Monash University and Alfred Health Melbourne Victoria Australia; ^16^ Department of Dermatology St George Hospital and Faculty of Medicine, University of New South Wales Sydney New South Wales Australia

**Keywords:** birch triterpenes/birch bark extract/Oleogel‐S10, dressing change burden, epidermolysis bullosa, pediatric, wound healing

## Abstract

**Background/Objectives:**

Pediatric patients with epidermolysis bullosa (EB) experience lifelong complications, and wound healing is an important treatment goal. In the phase III EASE study (NCT03068780), Oleogel‐S10 accelerated wound healing in EB. This prespecified subgroup analysis evaluated the long‐term efficacy and safety of Oleogel‐S10 in patients aged < 18 years from EASE.

**Methods:**

EASE comprised a 90‐day double‐blind phase (DBP) and 24‐month open‐label phase (OLP). In the DBP, patients were randomized 1:1 to receive Oleogel‐S10 or control gel; all patients could enter the OLP and receive Oleogel‐S10. Endpoints in this analysis included wound healing, total body wound burden, dressing change burden, and safety.

**Results:**

Overall, 156 pediatric patients were enrolled into the DBP (Oleogel‐S10: *n* = 74; Control gel: *n* = 82); 147 entered the OLP. Oleogel‐S10 resulted in 44.6% of pediatric patients with first complete target wound closure within 45 days, versus 25.6% with control gel (relative risk [95% CI]: 1.70 [1.11, 2.60], *p* = 0.012). Mean absolute body surface area percentage scores were 12.5% at DBP baseline and 5.3% at OLP Month 24 for the Oleogel‐S10 group. Significantly more patients treated with Oleogel‐S10 (38%) no longer required daily dressing changes versus control gel (9%) at DBP Day 90. Similar proportions of patients experienced adverse events in the Oleogel‐S10 and control gel groups in the DBP, with most mild or moderate.

**Conclusions:**

These analyses of pediatric patients demonstrated accelerated wound healing, reduced frequency of dressing changes, and sustained reduction in wound burden up to 27 months. Long‐term safety was consistent with the overall EASE population.

**Trial Registration:** NCT03068780, EudraCT 2016‐002066‐32; registered 3 March 2017; https://clinicaltrials.gov/study/NCT03068780 and https://www.clinicaltrialsregister.eu/ctr‐search/trial/2016‐002066‐32/GB

## 1 Introduction

Epidermolysis bullosa (EB) is a heterogeneous group of rare, inherited, skin conditions characterized by blistering of the skin that usually occurs in response to minor trauma [1]. Impaired wound healing predisposes patients to serious and potentially fatal complications, deformities, and symptoms [2]. Pediatric patients with EB experience unique management challenges and lifelong complications that have a devastating impact on daily life for patients and their families [3]. These children require consistent wound care management and frequent dressing changes that are often associated with significant pain [3]. Therapies that target wound healing and control inflammation could help address these severe and debilitating disease manifestations [2].

Oleogel‐S10 gel, also known as birch triterpenes/birch bark extract, is indicated for the treatment of wounds associated with dystrophic (DEB) and junctional EB (JEB) in adult and pediatric patients ≥ 6 months of age [4, 5]. Although three therapies are now approved by the United States Food and Drug Administration (FDA) for the treatment of DEB, Oleogel‐S10 is currently the only approved treatment in both DEB and JEB [5, 6, 7]. The pivotal phase III EASE study (NCT03068780, EudraCT 2016‐002066‐32) demonstrated accelerated wound healing with Oleogel‐S10 treatment, with long‐term safety and efficacy shown in the open‐label phase (OLP) [8, 9]. A significant benefit of Oleogel‐S10 treatment was observed in patients with recessive DEB (RDEB) and those aged < 18 years [8, 10].

The aim of this prespecified subgroup analysis was to evaluate the efficacy and safety of Oleogel‐S10 treatment in the subgroup of patients with EB aged < 18 years from the EASE study, and to further understand the disease burden for these patients.

## 2 Materials and Methods

### 2.1 Study Design

This was a prespecified subgroup analysis of the phase III EASE study population. The EASE study design was described previously [8, 9, 11]. Briefly, EASE was a randomized, controlled study, comprising a 90‐day double‐blind phase (DBP) and a 24‐month OLP, conducted from April 2017 to May 2022 (Supporting Information: Methods) [8, 9]. In the DBP, patients aged ≥ 21 days with DEB, JEB or Kindler EB (KEB) were randomized 1:1 to receive treatment with Oleogel‐S10 gel or a vehicle control gel (termed control gel) on all partial‐thickness wounds, with one target wound selected by the Investigator for each patient [8]. Following the DBP, all patients were eligible to enter the single‐arm OLP for treatment with Oleogel‐S10 on all EB partial‐thickness wounds. Patients were grouped based on their initial treatment in the DBP (Oleogel‐S10 group or former control gel group) [9].

### 2.2 Outcomes and Assessments

This prespecified subgroup analysis included patients < 18 years old with the following subgroups: those aged 0–< 4 years, 4–< 12 years, and 12–< 18 years at DBP baseline. Endpoints were first complete target wound closure by Day 45 ± 7 and time to first complete target wound closure by Day 90 ± 7. Additional wound‐related endpoints included total body wound burden, measured using body surface area percentage (BSAP [12]) affected by EB partial‐thickness wounds and the skin activity section of the EB disease activity and scarring index (EBDASI [13]), from DBP baseline up to OLP Month 24. Patient‐reported outcomes included procedural pain and dressing change burden up to Day 90, and Oleogel‐S10 tube usage up to OLP Month 24. Safety endpoints included incidence, severity, and relatedness of adverse events (AEs) up to OLP Month 24.

### 2.3 Statistical Analysis

For this prespecified subgroup analysis, in line with the overall population analysis, statistical analysis of the proportion of patients with first complete target wound closure by Day 45 was conducted using a Cochran–Mantel–Haenszel (CMH) test and stratified by EB subtype and target wound size class. Time to first complete target wound closure was analyzed using a non‐stratified Log‐Rank test. Pre‐planned analyses, per the statistical analysis plan, for the mean changes from baseline in BSAP and EBDASI were performed using an analysis of covariance (ANCOVA) with treatment group, EB subtype, and target wound size at baseline as fixed effects and BSAP/EBDASI total activity score at baseline as a covariate. BSAP and EBDASI assessments recorded within OLP visit windows were lower than expected, largely due to COVID‐19. Therefore, an additional analysis was produced without visit windows in the OLP, at the time of the study analysis. Data on change in frequency of dressing changes were analyzed post hoc, with patients no longer requiring daily dressing changes in the DBP analyzed using an ANCOVA. All other data presented are descriptive. Comparisons were only made between the randomized Oleogel‐S10 and control gel groups in the DBP. No comparisons between groups in the OLP could be made.

## 3 Results

### 3.1 Baseline Demographics and Patient Characteristics

Overall, 156 pediatric patients were enrolled in the DBP: 74 patients in the Oleogel‐S10 group and 82 patients in the control gel group. Of these patients, 147 entered the OLP. Baseline demographics and characteristics for the pediatric population were largely similar across the DBP and OLP (Table 1). At DBP baseline, median (range) age was 9.5 (0.5–17) years, and the majority of patients were aged 4–< 12 years (54.5%). More than 80% of patients in both treatment groups had RDEB. No patients with KEB were enrolled. While most target wounds measured 10–< 20 cm^2^, a substantial proportion (> 30%) were ≥ 20 cm^2^. The median age of target wounds was 38.0 days (range: 21–4745) for both treatment groups.

**TABLE 1:** Baseline patient demographics and characteristics.

	DBP		OLP*		
	Oleogel‐S10 (*n* = 74)	Control gel (*n* = 82)	Oleogel‐S10 (*n* = 69)	Former control gel (*n* = 78)	All patients (*n* = 147)
Age groups, *n* (%)					
0–< 4 years	7 (9.5)	10 (12.2)	7 (10.1)	9 (11.5)	16 (10.9)
4–< 12 years	42 (56.8)	43 (52.4)	40 (58.0)	41 (52.6)	81 (55.1)
12–< 18 years	25 (33.8)	29 (35.4)	22 (31.9)	28 (35.9)	50 (34.0)
Age, years					
Mean (95% CI)	9.6 (8.5–10.7)	9.1 (8.1–10.1)	9.4 (8.3–10.5)	9.2 (8.1–10.2)	9.3 (8.5–10.0)
Median (range)	10 (1–17)	9 (0–17)	10 (1–17)	9 (0–17)	10 (0–17)
EB subtype^§^					
Recessive DEB, *n* (%)					
Generalized severe	45 (60.8)	52 (63.4)	42 (60.9)	49 (62.8)	91 (61.9)
Generalized intermediate	16 (21.6)	11 (13.4)	15 (21.7)	10 (12.8)	25 (17.0)
Localized/other	4 (5.4)	5 (6.1)	4 (5.8)	5 (6.4)	9 (6.1)
Dominant DEB, *n* (%)	3 (4.1)	5 (6.1)	3 (4.3)	5 (6.4)	8 (5.4)
JEB, *n* (%)					
Generalized severe	0 (0.0)	1 (1.2)	0 (0.0)	1 (1.3)	1 (0.7)
Generalized intermediate	4 (5.4)	4 (4.9)	4 (5.8)	4 (5.1)	8 (5.4)
Localized/other	2 (2.7)	3 (3.7)^†^	1 (1.4)^‡^	3 (3.8)^†^	4 (2.7)
Target wound size group, *n* (%)					
10–< 20 cm^2^	50 (67.6)	54 (65.9)	47 (68.1)	53 (67.9)	100 (68.0)
20–< 30 cm^2^	14 (18.9)	17 (20.7)	13 (18.8)	14 (17.9)	27 (18.4)
30–50 cm^2^	10 (13.5)	11 (13.4)	9 (13.0)	11 (14.1)	20 (13.6)
Target wound size, cm^2^					
Mean (95% CI)	18.3 (16.4–20.2)	19.8 (17.6–22.0)	18.1 (16.3–20.0)	19.8 (17.5–22.1)	19.0 (17.5–20.5)
Median (range)	15.2 (10.0–43.7)	15.9 (10.0–49.5)	15.5 (10.0–37.0)	15.7 (10.0–49.5)	15.6 (10.0–49.5)
Age of target wound, days					
Mean (95% CI)	92.0 (57.4–126.6)	152.5 (34.4–270.6)	95.7 (58.7–132.7)	158.0 (33.9–282.2)	128.8 (61.2–196.4)
Median (range)	38.5 (21–950)	36.5 (21–4745)	39.0 (21–950)	36.5 (21–4745)	38.0 (21–4745)
BMI group, *n* (%)					
Underweight	41 (55.4)	42 (51.2)	38 (55.1)	40 (51.3)	78 (53.1)
Normal	30 (40.5)	34 (41.5)	28 (40.6)	32 (41.0)	60 (40.8)
Overweight	3 (4.1)	3 (3.7)	3 (4.3)	3 (3.8)	6 (4.1)
Obese	0 (0.0)	3 (3.7)	0 (0.0)	3 (3.8)	3 (2.0)
Mean BSAP (95% CI)	12.5 (10.2–14.8)	11.8 (9.5–14.0)	8.0 (6.5–9.4)	8.7 (6.9–10.6)	8.4 (7.2–9.5)
Mean EBDASI skin activity score (95% CI)	20.5 (17.8–23.3)	20.4 (17.6–23.2)	17.1 (14.7–19.6)	16.8 (14.2–19.4)	17.0 (15.2–18.7)

*Note:* *Data were reassessed at Day 90/OLP baseline. ^†^Only includes patients with JEB “other.” ^‡^Only includes JEB localized. ^§^One patient with EB simplex (EBS) was enrolled and included in this analysis; EBS patients were later excluded in a subsequent protocol amendment. This patient was randomized to the control gel group in the DBP and continued on to the OLP.

Abbreviations: BSAP, body surface area percentage; CI, confidence interval; DBP, double‐blind phase; DEB, dystrophic EB; EB, epidermolysis bullosa; EBDASI, EB disease activity and scarring index; EBS, EB simplex; JEB, junctional EB; OLP, open‐label phase.

### 3.2 First Complete Target Wound Closure

Oleogel‐S10 resulted in 44.6% of pediatric patients with first complete target wound closure within 45 days, compared with 25.6% in the control gel group (relative risk [95% CI]: 1.70 [1.11, 2.60], *p* = 0.012) (Figure 1A). For all age subgroups, a higher proportion of patients treated with Oleogel‐S10 experienced first complete target wound closure by Day 45 compared to control gel (Figure 1A), with significant differences reported between groups for patients aged 4–< 12 years (*p* = 0.021). For wounds that closed by Day 90, mean (95% CI) time to first complete target wound closure was 30.4 (24.5–36.3) days and 45.8 (37.01–54.6) days for the Oleogel‐S10 and control gel groups, respectively (Figure 1B). Images of target wound closure in patients from each age subgroup are shown in Figure S1.

**FIGURE 1:**
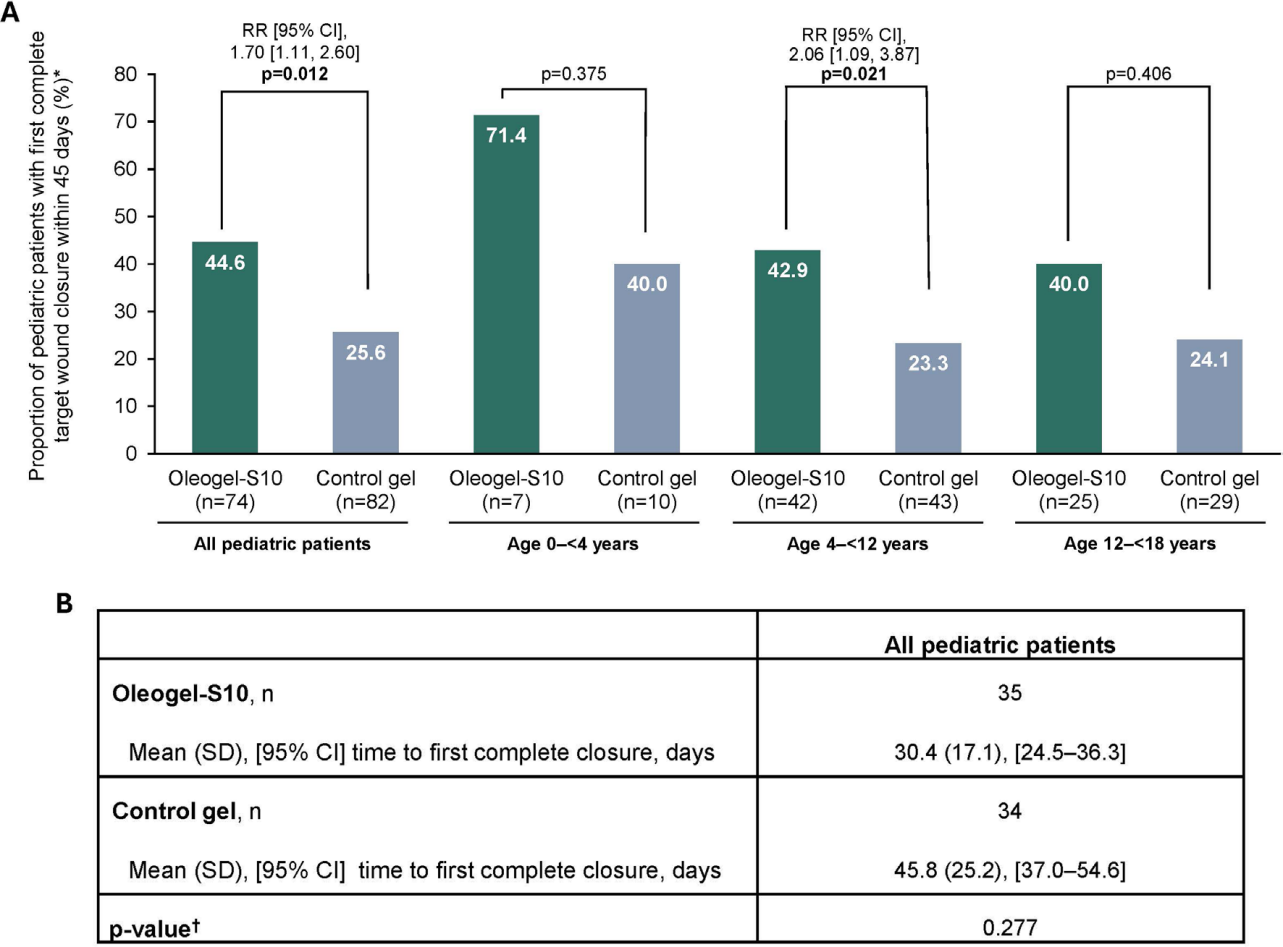
Proportion of pediatric patients with (A) first complete target wound closure by Day 45 and (B) time to first complete target wound closure up to Day 90. *Cochran–Mantel–Haenszel test stratified by EB subtype and target wound size class. ^†^Non‐stratified Log‐Rank test. CI, confidence interval; EB, epidermolysis bullosa; RR, relative risk; SD, standard deviation.

### 3.3 Total Wound Burden

Significant reductions in BSAP from DBP baseline were observed for the Oleogel‐S10 group across all timepoints in the DBP and OLP (Table S1). Mean (SD) change from DBP baseline to OLP Month 24 for the Oleogel‐S10 group was −5.1% (8.4) (*p* = 0.0005). Significant reductions were also observed for former control gel patients (mean [SD] change from DBP baseline to OLP Month 24: −3.0% [7.9], *p* = 0.0092). Mean absolute BSAP scores were 12.5% and 11.8% at DBP baseline and 5.3% and 6.2% at OLP Month 24 for the Oleogel‐S10 and former control gel groups, respectively (Figure 2A). Similar observations were made for EBDASI (Figure 2B, Table S2).

**FIGURE 2:**
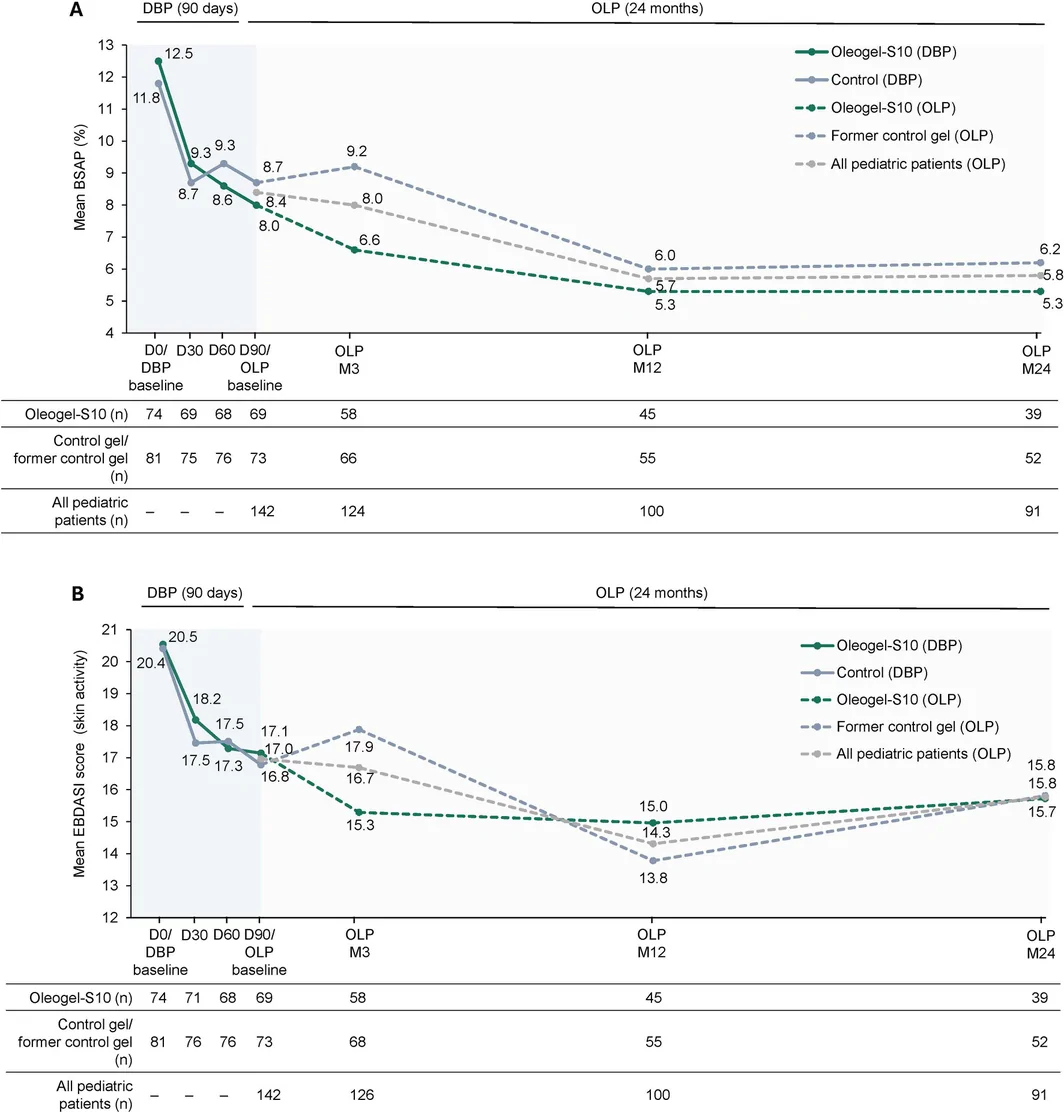
Mean BSAP (A) and EBDASI skin activity score (B) from DBP baseline to OLP Month 24 for pediatric patients. Data shown are the absolute mean BSAP and EBDASI skin activity score values at each timepoint. Assessments recorded within OLP visit windows were lower than expected largely due to COVID‐19. Therefore, a post hoc analysis was produced without visit windows in the OLP. Day 90/OLP baseline: The last assessment before the first open‐label treatment and at Day 90. Refer to Tables S1 and S2 for changes from DBP baseline data and statistical analysis. BSAP, body surface area percentage; D, Day; DBP, double‐blind phase; EBDASI, Epidermolysis Bullosa Disease Activity and Scarring Index; M, month; OLP, open‐label phase.

### 3.4 Patient‐Reported Outcomes

During the DBP, the number of patients no longer requiring daily dressing changes at Day 90 was significantly higher for patients treated with Oleogel‐S10 versus control gel (38% versus 9%, respectively) (Figure 3A). These reductions equated to three fewer dressing changes every 2 weeks for patients treated with Oleogel‐S10 versus less than one fewer dressing change for control gel. Change from baseline in mean time required for dressing changes up to Day 90 is shown in Figure 3B. Procedural pain was reduced at Day 90 for all age subgroups treated with Oleogel‐S10 (Figure S2). The median number of Oleogel‐S10 tubes used per patient per month was lower in the OLP than in the DBP, with the greatest reduction observed in patients aged 4–< 12 years (24.1 versus 15.0 tubes in the DBP versus OLP, respectively) (Figure S3).

**FIGURE 3:**
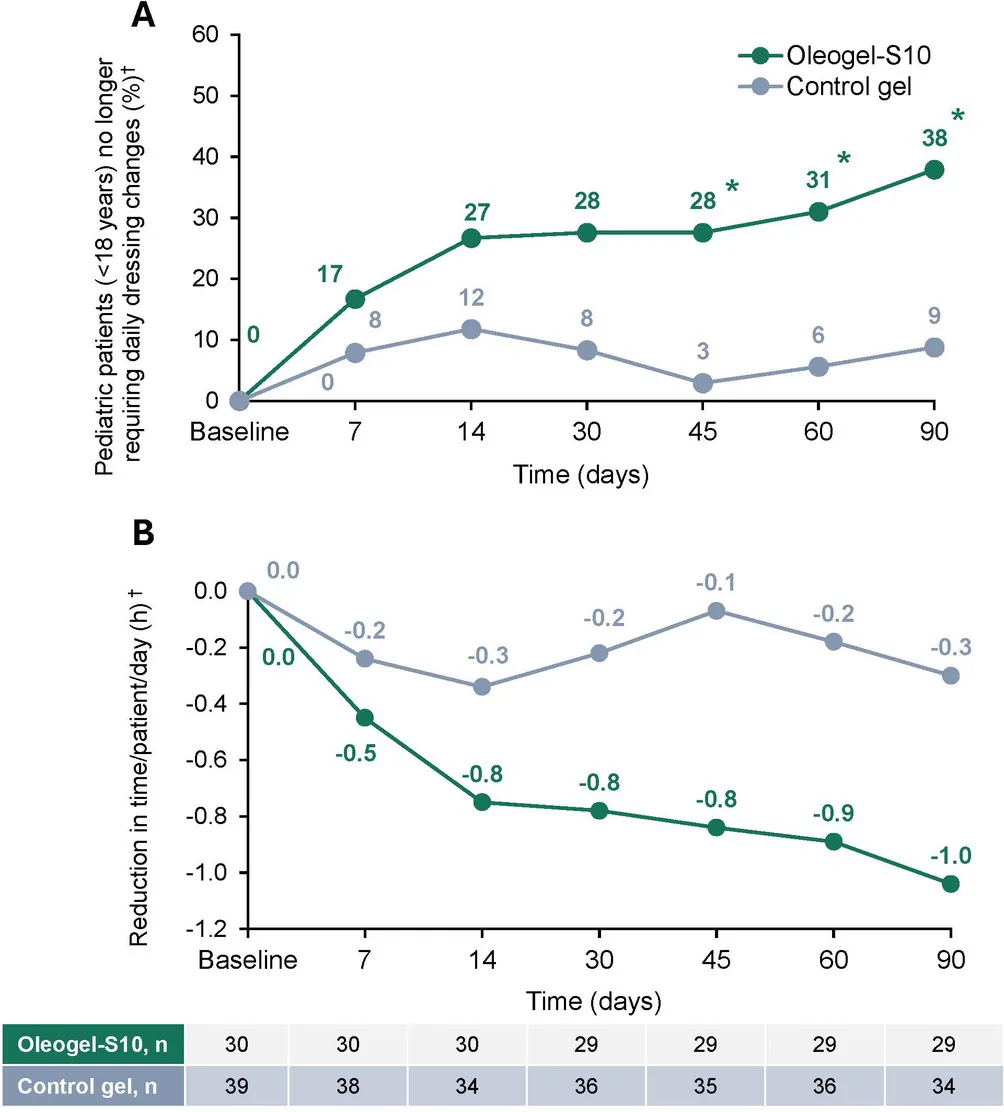
For pediatric patients that required daily dressing changes at baseline (A) those no longer requiring daily dressing changes and (B) reduction in time required for dressing changes per pediatric patient. *Oleogel‐S10 versus control gel *p* value < 0.05. ^†^Data derived from the subgroup of pediatric patients requiring daily dressing changes at baseline. Mean time spent per dressing change at baseline for both Oleogel‐S10 and control gel groups was 2.7 h.

### 3.5 Safety and Tolerability

AEs in pediatric patients are shown in Table 2. The majority of pediatric patients experienced an AE, with similar proportions observed between Oleogel‐S10 and control gel groups in the DBP (83.8% versus 81.7%, respectively). Most AEs were mild or moderate in severity, and the most frequent AE was wound complication. The proportions of patients experiencing a serious or treatment‐related AE were also similar between groups in the DBP. Overall, despite the longer observation period, fewer patients experienced an AE during the OLP (76.2% of all patients) than DBP (83.8% and 81.7% for the Oleogel‐S10 and control gel groups, respectively); fewer patients also experienced a treatment‐related AE in the OLP (12.2% of all patients versus 25.7% and 24.4% for the Oleogel‐S10 and control gel groups in the DBP, respectively). Few patients reported study or treatment withdrawal due to an AE in either the DBP or OLP. Five patients experienced a serious AE leading to death in the OLP, all occurring in the Oleogel‐S10 treatment group; none were related to treatment. Reasons for death were EB progression, sepsis, cardiac failure, cardio‐respiratory arrest, and pneumonia.

**TABLE 2:** Summary of AEs for pediatric patients.

	DBP		OLP		
	Oleogel‐S10 (*n* = 74)	Control gel (*n* = 82)	Oleogel‐S10 (*n* = 69)	Former control gel (*n* = 78)	All patients (*n* = 147)
Number of AEs, *n*	207	225	320	353	673
Patients with any AEs, *n* (%)	62 (83.8)	67 (81.7)	52 (75.4)	60 (76.9)	112 (76.2)
Leading to treatment withdrawal	2 (2.7)	2 (2.4)	5 (7.2)	5 (6.4)	10 (6.8)
Leading to study withdrawal	2 (2.7)	0 (0.0)	5 (7.2)	5 (6.4)	10 (6.8)
Most frequent AEs (> 10% of patients in any group)					
Wound complication*	47 (63.5)	47 (57.3)	25 (36.2)	35 (44.9)	60 (40.8)
Pyrexia	8 (10.8)	13 (15.9)	8 (11.6)	9 (11.5)	17 (11.6)
Pruritus	8 (10.8)	4 (4.9)	4 (5.8)	7 (9.0)	11 (7.5)
Wound infection	5 (6.8)	5 (6.1)	5 (7.2)	10 (12.8)	15 (10.2)
Anemia	5 (6.8)	4 (4.9)	13 (18.8)	17 (21.8)	30 (20.4)
Wound infection staphylococcal	3 (4.1)	3 (3.7)	9 (13.0)	12 (15.4)	21 (14.3)
Esophageal stenosis	0	1 (1.2)	6 (8.7)	10 (12.8)	16 (10.9)
Wound infection bacterial	2 (2.7)	3 (3.7)	7 (10.1)	7 (9.0)	14 (9.5)
Patients with any serious AEs	5 (6.8)	4 (4.9)	18 (26.1)	21 (26.9)	39 (26.5)
Leading to study withdrawal	1 (1.4)	0 (0.0)	5 (7.2)	2 (2.6)	7 (4.8)
Leading to death*	0 (0.0)	0 (0.0)	5 (7.2)	0 (0.0)	5 (3.4)
Most frequent serious AEs (> 5% of patients in any group)					
Anemia	3 (4.1)	0 (0.0)	4 (5.8)	6 (7.7)	10 (6.8)
Esophageal stenosis	0 (0.0)	0 (0.0)	3 (4.3)	6 (7.7)	9 (6.1)
Patients with any severe AEs	9 (12.2)	3 (3.7)	14 (20.3)	15 (19.2)	29 (19.7)
Most frequent severe AEs (> 5% of patients in any group)					
Anemia	4 (5.4)	0 (0.0)	3 (4.3)	5 (6.4)	8 (5.4)
Esophageal stenosis	0 (0.0)	0 (0.0)	2 (2.9)	4 (5.1)	6 (4.1)
Patients with any treatment‐related AEs	19 (25.7)	20 (24.4)	6 (8.7)	12 (15.4)	18 (12.2)
Leading to study withdrawal	2 (2.7)	0 (0.0)	0 (0.0)	3 (3.8)	3 (2.0)
Most frequent treatment‐related AEs (> 5% of patients in any group)					
Wound complication^†^	11 (14.9)	12 (14.6)	3 (4.3)	8 (10.3)	11 (7.5)
Wound infection staphylococcal	1 (1.4)	2 (2.4)	1 (1.4)	4 (5.1)	5 (3.4)
Patients with any serious treatment‐related AEs	1 (1.4)	0 (0.0)	1 (1.4)	1 (1.3)	2 (1.4)
Leading to study withdrawal	1 (1.4)	0 (0.0)	0 (0.0)	1 (1.3)	1 (0.7)

*Note:* AEs presented are those defined as treatment emergent (from first application to 30 days post last application). An additional five non‐treatment‐emergent AEs occurred, all unrelated to treatment and with an onset date > 30 days after last date of study medication administration in the OLP. Three were serious: one patient was in the former control gel group who died of sepsis, and two patients were in the Oleogel‐S10 treatment group (oral disorder and gastrostomy tube site complication). AEs were coded with MedDRA Version 23.0. *Serious AEs leading to death occurred in four patients with generalized‐severe RDEB and one patient with RDEB (other). All serious AEs leading to death were judged independently by the reporting Investigator and the Sponsor as unlikely to be related to treatment. Causes of death were: disease progression (study Day 170), sepsis (study Day 355, consistent with underlying EB disease), cardiac failure (study Day 379, assessed as likely due to inadequately treated anemia and hypoalbuminemia due to EB by the Independent Data Safety Monitoring Committee), pneumonia (study Day 761, consistent with underlying EB disease), and cardio‐respiratory arrest (study Day 851, 29 days after end of study, likely due to previous serious AEs including COVID‐19 infection, acute gastrointestinal bleeding, and hypovolemic shock). These causes of death align with published registry data for early comorbidities (such as anemia, failure to thrive, inflammation) [14] contributing to the most frequently reported causes (e.g., pneumonia, respiratory failure, sepsis) of childhood death in EB [15]. ^†^Wound complication encompassed the following events: increase in wound size versus baseline (i.e., baseline of the DBP); wound reopening; increase in wound size versus the previous visit; other (including increase in wound burden, worsening of epidermolysis bullosa wound pain, and wound odor); injury to the wound; wound worsening versus baseline (i.e., baseline of the DBP).

Abbreviations: AE, adverse event; DBP, double‐blind phase; MedDRA, Medical Dictionary for Regulatory Activities; OLP, open‐label phase.

## 4 Discussion

This prespecified subgroup analysis of pediatric DEB and JEB patients from the pivotal phase III EASE study provides important long‐term efficacy and safety data for Oleogel‐S10 treatment up to 27 months. Due to the complications that can develop in children with EB, the importance of effective wound management from an early age cannot be understated [3, 16, 17]. Wounds are often recurrent, worsened by infections or chronic inflammation, leading to non‐healing wounds [18]. Specifically for children with EB, effective wound healing could help improve daily life by preventing infection, shortening wound duration, reducing overall wound burden, and potentially modifying morbidity associated with wounding and fibrosis, such as pseudosyndactyly [17]. Consistent with the overall EASE population [8, 9], Oleogel‐S10 accelerated wound healing and reduced the extent of open wounds in pediatric patients. Reductions in wound burden (measured using BSAP and EBDASI) were also sustained for up to 2 years over the OLP, which may be clinically meaningful in the context of a disease with a progressive natural history.

Treatments that can effectively reduce the frequency of dressing changes are urgently needed due to the distressing and time‐consuming nature of this procedure for patients with EB, and the significant impact this has on their caregivers and families [3]. A greater proportion of pediatric patients treated with Oleogel‐S10 no longer required daily dressing changes at Day 90 compared to those treated with control gel. This reduction in dressing changes saved patients and caregivers combined an estimated ~12 h per week when treated with Oleogel‐S10 versus ~4 h with control gel at Day 90, which is consistent with results from the overall EASE population [19]. This calculation is based on published evidence for the time required for whole body wound care; an additional 66.7% of time spent by caregivers was added to the patient time [3, 19].

Dressing changes can cause significant pain [20], with patients often requiring potent analgesics (such as opioids) and anxiolytics to palliate symptoms [21]. Improvements in procedural pain across all pediatric age subgroups were observed for patients treated with Oleogel‐S10 in the DBP. This improvement may reduce the need for potent analgesics, which are often associated with AEs. The number of Oleogel‐S10 tubes used by patients per month was also reduced in the OLP compared to the DBP, with the greatest reduction observed in the age 4–< 12 years subgroup, supporting the observed reductions in BSAP and dressing change frequency. Data on patient‐reported outcomes in EB are limited; our subgroup analysis provides important insights into these outcomes in young patients.

The safety profile of Oleogel‐S10 in this subgroup analysis was consistent with the overall EASE population [8, 9], with no safety concerns observed for pediatric patients. The majority of pediatric patients experienced an AE in the DBP, with most mild or moderate and similar proportions observed between groups. The proportion of patients experiencing any AE in the OLP was lower than in the DBP, including those due to wound complications or that were treatment‐related, suggesting no safety concerns with longer term treatment of Oleogel‐S10. Overall, few patients experienced AEs that led to treatment or study withdrawal, suggesting that Oleogel‐S10 is well‐tolerated in this population.

The limitations of this analysis include the low number of patients in each age subgroup, particularly the age 0–< 4 years subgroup. Due to low patient numbers, not all results were shown by age subgroup to provide sufficient patient numbers for meaningful interpretation. The open‐label study design was chosen to minimize time on the control gel, which is ethically important in rare, severe diseases.

## 5 Conclusion

This prespecified subgroup analysis provides the most robust long‐term data to date for Oleogel‐S10 in pediatric EB. Oleogel‐S10 was associated with faster wound healing, sustained reductions in wound burden, and fewer dressing changes, leading to meaningful improvements in daily care and reducing the burden on patients and caregivers. Long‐term safety data were consistent with that observed for the overall EASE population, and Oleogel‐S10 was well‐tolerated in pediatric patients. These findings establish Oleogel‐S10 as a valuable therapeutic option for pediatric EB and underscore the importance of ongoing real‐world data to determine how early treatment may influence disease progression and the risk of severe complications in this vulnerable population.

## Author Contributions

E.S., M.T‐.P., M.F.F., C.B., A.L.B., S.M.d.M.B., A.D., A.E.M., F.P., M.N., A.V., R.d.L., J.S.K., and D.F.M. contributed to the conceptualization, writing – original draft, and writing – reviewing and editing. N.T.T. and S.L. contributed to the data curation, formal analysis, validation, writing – original draft, and writing – reviewing and editing.

## Funding

EASE was funded by Amryt Research Ltd., Dublin, Ireland (acquired by Chiesi Farmaceutici S.p.A. April 2023). Trial registration: NCT03068780, EudraCT 2016‐002066‐32; registered 3 March 2017; https://clinicaltrials.gov/study/NCT03068780 and https://www.clinicaltrialsregister.eu/ctr‐search/trial/2016‐002066‐32/GB.

## Ethics Statement

EASE was conducted in accordance with the Declaration of Helsinki, and the research protocol was approved by the institutional review boards and ethics committees for each study site. All patients provided written informed consent.

## Conflicts of Interest

M.T‐.P. reports grants/fees from AbbVie, Amryt Pharma, Avicanna, Chiesi, Eucerin, Galderma, Gencell Pharma, Lilly, Novartis, Pfizer, Pierre Fabre, Rheacell, Roemmers, Sanofi and Uno Healthcare. M.F.F. and C.B. report personal fees from Amryt during the conduct of the study. A.L.B. reports grants and other support from Amryt during the conduct of the study; grants and other support from Amicus/Scioderm, other support from Chiesi and Krystal Biotech, grants and other support from Fibrocell/Castle Creek, and grants from EB Research Partnership and EB Medical Research Foundation, Phoenix Tissue Repair, ProQR/Wings and Rheacell outside the submitted work. A.D. reports personal fees from Amryt during the conduct of the study. A.E.M. has been involved in previous and current clinical trials with Amryt Pharma, Rheacell GmbH & Co. KG and TWi Biotechnology, and has served as a consultant for KrystalBio. F.P. was a Principal Investigator for Amryt during the conduct of the study. M.N. reports personal fees from Amryt during the conduct of the study and other fees from AbbVie, AstraZeneca, Eli Lilly, Janssen/Johnson & Johnson, Novartis, Takeda and UCB. A.V. was a Principal Investigator for Amryt during the conduct of the study and reports other fees from AbbVie, Almirall, Amgen, Amryt Pharma, Boehringer, Bristol Myers Squibb, Galderma, Jansen, LeoPharma, Lilly, Incyte, Novartis, Pfizer, Sanofi, and UCB. R.d.L. was a Principal Investigator for Amryt during the conduct of the study. N.T.T. and S.L. are employees of Chiesi Farmaceutici S.p.A. J.S.K. reports grants to institution from Amryt/Chiesi during the conduct of the study and other grants to institution from AbbVie, Amicus, Amgen, Arcutis, Argenix, Astra‐Zeneca, Biofrontera, Boehringer, BMS, Celldex, CSL, DFG/Fresenius, Dompé, Eli Lilly, Exopharm, Galderma, Genentech, Incyte, InflaRx, Johnson & Johnson, Kiniksa, Merck KGaA, Mitsubishi, Nektar, Novartis, Pfizer, Regeneron, Roche, Sanofi, Takeda and UCB outside the submitted work. D.F.M. reports personal fees from Amryt during the conduct of the study and other fees from AbbVie, Amicus, Amgen, Amryt Pharma, Arcutis, Arenan, Argenix, AstraZeneca, BMS, Castle Creek, Cutanea, CSL, Dermira, Eli Lilly, Evelo, Galderma, Genentech, Incyte, JCB Biopharma, Kiniksa, Novartis, Pfizer, Phoenix Tissue Repair, Pierre Fabre, Principia, ProQR/Wings, Regeneron, Rheacell, Sanofi, Scioderm, Shire, Takeda and UCB, and is a co‐patent holder with Stanford University for the use of topical sirolimus for epidermolysis bullosa simplex. The other authors declare no conflicts of interest.

## Supporting information


**Table S1:** Mean change from DBP baseline in BSAP for pediatric patients.
**Table S2:** Mean change from DBP baseline in EBDASI skin activity for pediatric patients.
**Figure S1:** Target wound closure in patients treated with Oleogel‐S10 aged (A and B) 0–< 4 years, (C and D) 4–< 12 years and (E and F)12–< 18 years.
**Figure S2:** Procedural pain for pediatric patients during the DBP.
**Figure S3:** Oleogel‐S10 tube usage for pediatric patients.

## Data Availability

Selected subgroup‐level data, figures and listings can be made available on request from the corresponding author (johannes.kern@monash.edu). These will be available from 1 year after completion of the trial and in perpetuity. The study protocol, statistical analysis plan and consent form can also be made available upon request via the corresponding author as per the original study data published in 2023 by Kern et al. [8].
