# Supplementary material for: Long‐Term Efficacy and Safety of Oleogel‐S10 (Birch Triterpenes) for Pediatric Patients With Epidermolysis Bullosa
Source: Pediatr Dermatol. 2026 May 28;43(5):1111–9. doi: 10.1111/pde.70224 (PMC13617162; doi:10.1111/pde.70224)
Supplement: Supplementary file 1 [file PDE-43-1111-s001.pdf]

SUPPLEMENTARY MATERIAL

MATERIALS AND METHODS

EASE study design

Study gel was applied at every dressing change (at least every four days) to all partial-thickness wounds. The patient could keep their usual established schedule of dressing changes, provided dressing changes and study medication application occurred at least every 4 days. The patient/caregiver was asked to report their schedule of dressing changes at each clinic visit. Target wounds were between 10–50 cm^2 in size and present for ≥21 days and <nine months (1). In the OLP, patients were grouped based on those formerly treated with Oleogel-S10 in the DBP (Oleogel-S10 group) and those formerly treated with control gel in the DBP (former control gel group) (2). The patient groups in the OLP were not directly compared as these patients were receiving treatment with Oleogel-S10 for differing durations (2).

Outcomes and assessments

Complete wound closure was defined as skin re-epithelialization without drainage (3). Total EBDASI score was amended to 100 as anogenital regions and buttocks were excluded from examination (2). Procedural pain was measured using Face Legs Arms Cry Consolability score (FLAAC) (patients aged <4 years) and Wong Baker Faces (patients aged 4–<18 years) (3).

Table S1. Mean change from DBP baseline in BSAP for pediatric patients

	DBP		OLP		
	Day 0 / DBP baseline	Day 90 / OLP baseline	Month 3	Month 12	Month 24
**Oleogel-S10**, n	74	69	58	45	39
Mean (SD) change from DBP baseline	–	-4.7 (7.2)	-5.0 (8.2)	-6.9 (9.1)	-5.1 (8.4)
95% CI	–	-6.41, -2.96	-7.13, -2.81	-9.66, -4.17	-7.80, -2.37
p-value	–	<0.0001	<0.0001	<0.0001	0.0005
**Former control gel**, n	81	72	65	54	51
Mean (SD) change from DBP baseline	–	-2.1 (7.1)	-1.9 (10.6)	-3.9 (8.9)	-3.0 (7.9)
95% CI	–	-3.82, -0.46	-4.54, 0.72	-6.32, -1.49	-5.22, -0.78
p-value	–	0.0132	0.1522	0.0020	0.0092
**All pediatric patients**, n	155	141	123	99	90
Mean (SD) change from DBP baseline	–	-3.4 (7.2)	-3.4 (9.6)	-5.3 (9.1)	-3.9 (8.1)
95% CI	–	-4.59, -2.18	-5.07, -1.63	-7.08, -3.47	-5.60, -2.20
p-value	–	<0.0001	0.0002	<0.0001	<0.0001

Assessments recorded within OLP visit windows were lower than expected largely due to COVID-19. Therefore, a post hoc analysis was produced without visit windows in the OLP. BSAP, body surface area percentage; CI, confidence interval; DBP, double-blind phase; OLP, open-label phase; SD, standard deviation.

Table S2. Mean change from DBP baseline in EBDASI skin activity for pediatric patients

	DBP		OLP		
	**Day 0 / DBP baseline**	**Day 90 / OLP baseline**	**Month 3**	**Month 12**	**Month 24**
**Oleogel-S10**, n	74	69	58	45	39
Mean (SD) change from DBP baseline	–	-3.6 (8.7)	-5.0 (9.4)	-6.3 (8.7)	-4.4 (8.9)
95% CI	–	-5.69, -1.52	-7.49, -2.55	-8.89, -3.64	-7.27, -1.50
p-value	–	0.0010	0.0001	<0.0001	0.0038
**Former control gel**, n	81	72	67	54	51
Mean (SD) change from DBP baseline	–	-2.8 (7.2)	-2.1 (10.2)	-5.4 (9.2)	-2.8 (9.7)
95% CI	–	-4.48, -1.08	-4.56, 0.44	-7.90, -2.88	-5.48, -0.01
p-value	–	0.0017	0.1044	<0.0001	0.0491
**All pediatric patients**, n	155	141	125	99	90
Mean (SD) change from DBP baseline	–	-3.2 (8.0)	-3.4 (9.9)	-5.8 (9.0)	-3.5 (9.4)
95% CI	–	-4.51, -1.86	-5.19, -1.68	-7.58, -4.00	-5.41, -1.50
p-value	–	<0.0001	0.0002	<0.0001	0.0007

Assessments recorded within OLP visit windows were lower than expected largely due to COVID-19. Therefore, a post hoc analysis was produced without visit windows in the OLP. CI, confidence interval; DBP, double-blind phase; EBDASI, Epidermolysis Bullosa Disease Activity and Scarring Index; OLP, open-label phase; SD, standard deviation.

Figure S1. Target wound closure in patients treated with Oleogel-S10 aged (A,B) 0-<4 years, (C,D) 4-<12 years and (E,F)12-<18 years.

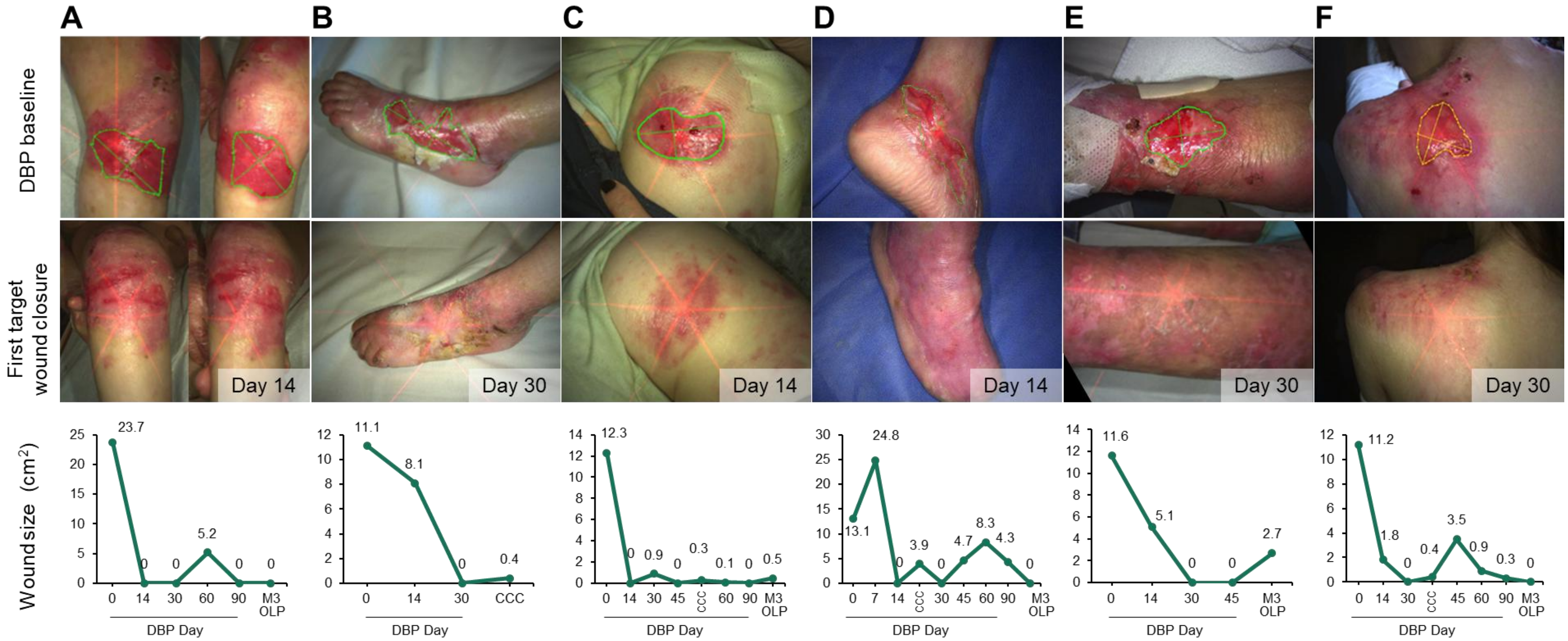

Patient	A	B	C	D	E	F
Patient age	2 years	3 years	7 years	11 years	16 years	17 years
EB type/subtype	RDEB, GS	RDEB, GI	RDEB, GS	RDEB, GI	RDEB, GS	RDEB, GS
Wound location	Right knee, anterior*	Left foot, dorsum	Right buttock, medial	Right foot, lateral	Left thigh, anterior	Left back, scapular region
Wound size at DBP baseline (cm²)	23.7	11.1	12.3	13.1	11.6	11.2
Wound age at DBP baseline (days)	21	60	28	22	60	72

Top panel: Representative images for each pediatric age subgroup showing first complete target wound closure in EB patients treated with Oleogel-S10. Middle panel: Corresponding target wound size graphs showing wound size (cm^2) from DBP baseline to month 3 OLP where evaluations were undertaken and available. *Patient A required two tracings to capture the full wound due to its location. CCC, confirmation of complete closure, which occurred 7 (+2) days after first clinical assessment of complete closure of the

EB target wound in the DBP. Note some patients did not have a CCC due to the visit performed outside of the permitted window or missed visits; DBP, double-blind phase; OLP, open-label phase; DDEB, dominant dystrophic EB; EB, epidermolysis bullosa; GI, generalized intermediate; GS, generalized severe; RDEB, recessive dystrophic EB.

Figure S2. Procedural pain for pediatric patients during the DBP

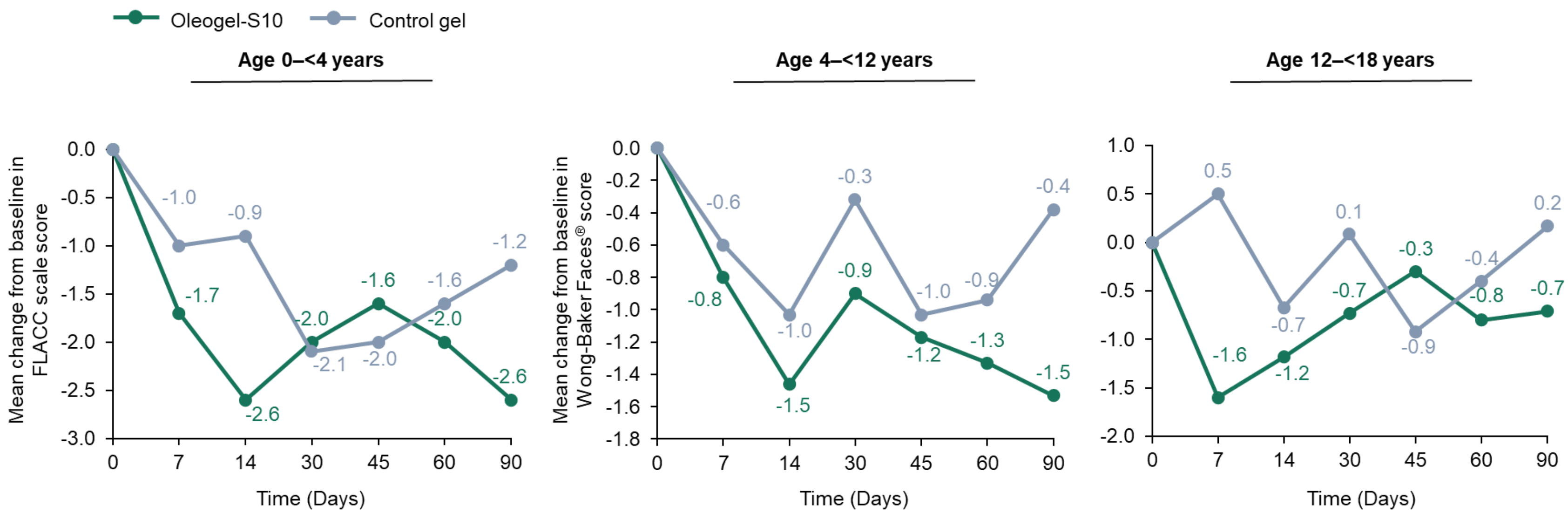

Oleogel-S10 (n)	7	3	7	7	5	7	7	41	15	37	40	36	39	34	24	9	22	22	20	20	17
Control gel (n)	10	3	9	8	8	8	6	41	14	39	38	37	36	32	28	15	27	26	24	25	24

DBP, double-blind phase; FLACC, Face Legs Arms Cry Consolability score.

Figure S3. Oleogel-S10 tube usage for pediatric patients

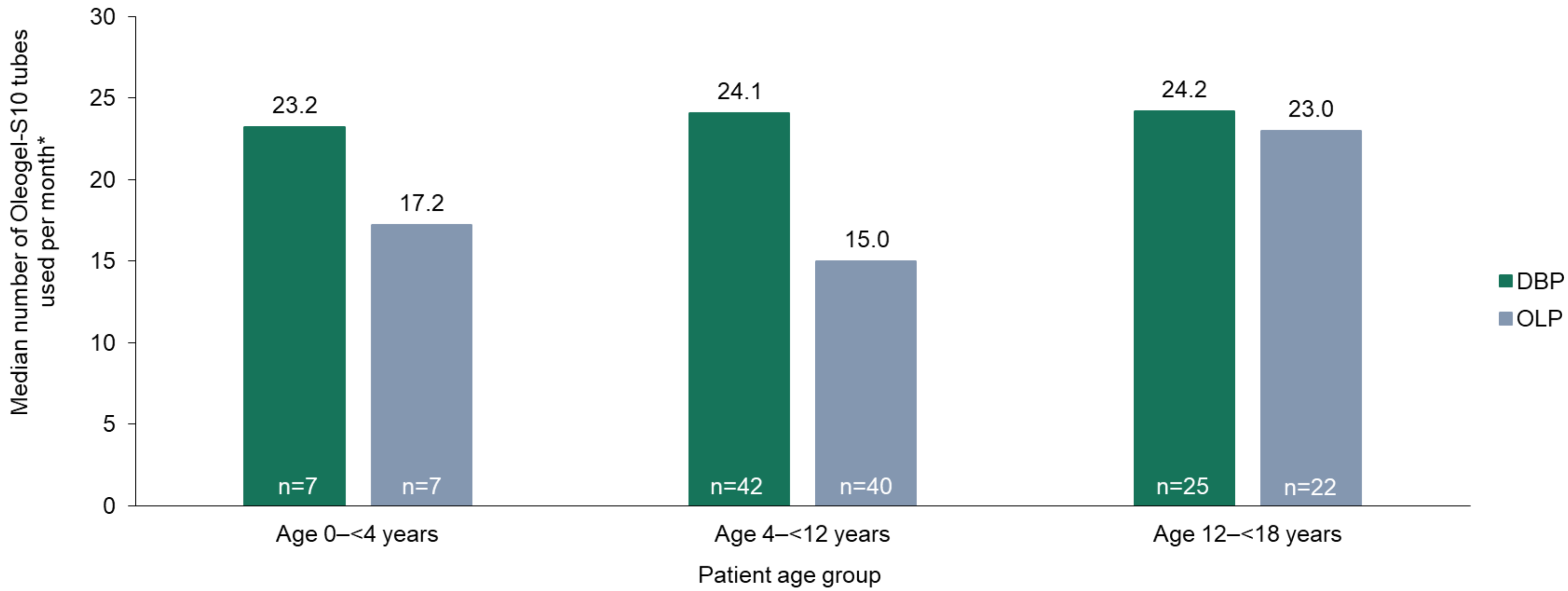

No statistical testing was performed. *n numbers were small for the 0–<4 years age group and these results should therefore be interpreted with caution. DBP, double-blind phase; OLP, open-label phase.

References

1. Kern JS, Schwieger-Briel A, Löwe S, Sumeray M, Davis C, Martinez AE. Oleogel-S10 Phase 3 study "EASE" for epidermolysis bullosa: study design and rationale. Trials 2019; 20(1):350.

2. Murrell DF, Bodemer C, Bruckner AL, Cunningham T, Davis C, Fernández MF et al. Long-term safety and efficacy of Oleogel-S10 (birch bark extract) in epidermolysis bullosa: 24-month results from the Phase III EASE Study. Br J Dermatol 2025; 192:1007–17.

3. Kern JS, Sprecher E, Fernandez MF, Schauer F, Bodemer C, Cunningham T et al. Efficacy and safety of Oleogel-S10 (birch triterpenes) for epidermolysis bullosa: results from the phase III randomized double-blind phase of the EASE study. Br J Dermatol 2023; 188(1):12–21.
